# Mitochondrial ROS Induce Partial Dedifferentiation of Human Mesothelioma via Upregulation of NANOG

**DOI:** 10.3390/antiox9070606

**Published:** 2020-07-10

**Authors:** Filip Sedlic, Fran Seiwerth, Ana Sepac, Suncana Sikiric, Marina Cindric, Marija Milavic, Lovorka Batelja Vuletic, Marko Jakopovic, Sven Seiwerth

**Affiliations:** 1 Department of Pathophysiology, University of Zagreb School of Medicine, 10 000 Zagreb, Croatia; 2 Department of Respiratory Diseases Jordanovac, University Hospital Centre Zagreb, 10 000 Zagreb, Croatia; fseiwerth@gmail.com (F.S.); marko.jakopovic@kbc-zagreb.hr (M.J.); 3 Department of Pathology, University of Zagreb School of Medicine, 10 000 Zagreb, Croatia; ana.sepac@mef.hr (A.S.); suncana.sikiric@mef.hr (S.S.); marija.milavic@mef.hr (M.M.); lovorka.batelja.vuletic@mef.hr (L.B.V.); sven.seiwerth@mef.hr (S.S.); 4 Clinical Department of Pathology and Cytology, University Hospital Center Zagreb, 10 000 Zagreb, Croatia; mcindric@kbc-zagreb.hr; 5 Department of Internal Medicine, University of Zagreb School of Medicine, 10 000 Zagreb, Croatia

**Keywords:** pluripotency factors, reprogramming, mesothelioma, reactive oxygen species, mitochondria

## Abstract

The expression of pluripotency factors is a key regulator of tumor differentiation status and cancer stem cells. The purpose of this study was to examine the expression of pluripotency factors and differentiation status of human mesothelioma and the role of mitochondria in their regulation. We tested the expression of OCT4/*POU5F1*, NANOG, SOX2, PI3K-AKT pathway and BCL2 genes and proteins in 65 samples of human mesothelioma and 19 samples of normal mesothelium. Mitochondrial membrane potential, reactive oxygen species (ROS) generation and expression of pluripotency factors were also tested in human mesothelioma cell line. Human mesothelium and mesothelioma expressed SOX2, NANOG, PI3K and AKT genes and proteins and *POU5F1* gene, whereby NANOG, SOX2 and phosphorylated (activated) AKT were upregulated in mesothelioma. NANOG protein expression was elevated in less differentiated samples of human mesothelioma. The expression of genes of PI3K-AKT pathway correlated with pluripotency factor genes. Mesothelioma cells had functional, but depolarized mitochondria with large capacity to generate ROS. Mitochondrial ROS upregulated NANOG and mitoTEMPO abrogated it. In conclusion, human mesothelioma displays enhanced expression of NANOG, SOX2 and phosphorylated AKT proteins, while elevated NANOG expression correlates with poor differentiation of human mesothelioma. Mitochondria of mesothelioma cells have a large capacity to form ROS and thereby upregulate NANOG, leading to dedifferentiation of mesothelioma.

## 1. Introduction

Malignant pleural mesothelioma originates from pleural mesothelial cells. It is notorious for its resistance to various types of therapy and poor survival. In spite of recent advances in understanding genetic identity of human mesothelioma, it remains unclear which molecular mechanisms drive its malignant behavior.

Activation of set of pluripotency genes such as *POU5F1*, *NANOG*, *SOX2* or *c-MYC* reprograms cells to undifferentiated state, giving them features that stem cells have during early development [1,2]. Cancer cells, especially cancer stem cells may recapitulate some of these features of undifferentiated cells, which could be responsible for local and distal spreading of the tumor, and even resistance to therapy [3]. Pluripotency factors, such as OCT4 or NANOG, are master regulators of dedifferentiation, and therefore may be critical for the clinical outcome of malignant tumors. For example, poor survival is associated with high expression of OCT4 (protein encoded by *POU5F1* gene) in gastric cancer [4], high expression of NANOG in lung [5] and breast cancer [6], or high expression of SOX2 in gastric cancer [7]. Moreover, high expression of OCT4 and NANOG correlates with resistance to cisplatin in squamous cell carcinoma [8]. Cancer stem cells exhibit high expression of OCT4, NANOG and SOX2, which represent their markers [9,10]. However, the expression of pluripotency factors in human mesothelioma and normal mesothelium is not completely investigated. Although Warburg effect suggest that mitochondria could be dispensable for viability of cancer cells, a growing body of evidence points to the crucial role of mitochondrial metabolism in cancer growth and metastasis [11]. Reactive oxygen species (ROS), produced by mitochondria regulate expression of numerous genes and cellular functions, including the expression of pluripotency genes and the differentiation status [12].

In many types of cancer, PI3K-AKT pathway drives important malignant characteristics, such as cell proliferation, survival, growth and metastasis [13]. It can upregulate anti-apoptotic BCL2, which could provide resistance to external stressors [14]. Pluripotency genes and PI3K-AKT pathway interact in a complex manner. PI3K-AKT pathway can induce OCT4 [15], NANOG [16] or stabilize SOX2 [17]. However, OCT4 and SOX2 can also lead to activation of PI3K-AKT pathway [18].

We designed this study to determine the expression of the most important pluripotency genes and proteins, OCT4, NANOG and SOX2 in human mesothelioma and to investigate its association with the PI3K-AKT-BCL2 pathway. Moreover, using the human mesothelioma cell line we also examined whether mitochondria-derived ROS drive the expression of these pluripotency factors, acting as potential regulators of mesothelioma dedifferentiation.

## 2. Materials and Methods

### 2.1. Human Mesothelium and Mesothelioma Samples

Immunohistochemical study included samples of 19 randomly selected normal pleuras and 65 cases of malignant pleural mesothelioma from the archives of the Department of Pathology, University of Zagreb School of Medicine and the Clinical Department of Pathology and Cytology, Clinical Hospital Center Zagreb. The study included the period between 2000 and 2018. There were 61 males and 4 females with an average age of 60 years. In order to avoid problems with possible long-term RNA instability, for PCR analysis we used 9 normal pleura controls, obtained by manual microdissection, and 34 mesothelioma samples diagnosed between 2016 and 2018. All experimental procedures were approved by the Institutional Ethical Committee (document number: 380-59-10106-15-168/265).

### 2.2. Cell Culture

Human mesothelioma cell line Mero-14 (The European Collection of Authenticated Cell Cultures) was cultured according to the manufacturer recommendation in the Ham F-10 (Merck) culture medium with 15% fetal calf serum (FCS, Merck) at 37 °C, in a humidified atmosphere containing 5% CO_2_. To stimulate mitochondrial ROS production, cells were treated with the mitochondrial electron transport chain complex III inhibitor antimycin A, and the mitoTEMPO was used to scavenge mitochondrial ROS.

### 2.3. Immunocyto(histo) Chemistry

Immunohistochemistry was used to determine the protein expression in human samples or Mero-14 cells. It was performed as we previously published [19]. Briefly, immunohistochemical detection was done using the EnVision Flex System (Dako, Denmark) at room temperature and the positive reaction was stained by the 3, 3-diaminobenzidine tetrachloride (DAB, Dako, Denmark). After fixation in the ice cold methanol, cells were treated with the peroxidase-blocking reagent for 5 min. This was followed by incubation with the diluted primary antibody at room temperature over a period of 1 h. The primary rabbit monoclonal antibodies were: anti-OCT4 (1/500, Abcam, ab200834), anti-NANOG (1/100, Abcam, ab109250), anti-PI3 Kinase p85 alpha (phospho Y607; 1/200, Abcam, ab182651) and anti-AKT1 (phospho S473; 1/200, Abcam, ab81283), while mouse monoclonal antibodies were: anti-SOX2 (1/200, Abcam, ab171380), anti-vimentin, clone Vim 3B4 (1/200, Agilent), anti-cytokeratin 7, clone OV-TL 12/30 (1/100, Agilent). Samples without primary antibodies served as negative controls. Appropriate tissues were used as positive controls for antibodies. Cells or fixed tissues were then incubated for 30 min in a secondary antibody (EnVision HRP, Agilent) and stained with the DAB for 1 min. The reaction was captured with the EVOS imaging system (Thermo Fisher Scientific) for cells or the camera mounted on the light microscope for tissue samples. The intensity of DAB staining in cells was determined using the ImageJ software (NIH), by subtracting light intensity over the cell (nucleus for SOX2 or cytosol for other proteins) from the light intensity of the background. Therefore, a greater value indicates greater expression of a protein. Immunohistochemical (Im) index, as semi-quantitative value, was used to analyze a protein expression in human tissue samples. It was determined by multiplying percentage of positively stained cells (0 = no positive cells; 1 = less than 10%; 2 = 10–50% and 3 = more than 50%) with the staining intensity (0 = no staining; 1 = weak; 2 = moderate and 3 = strong staining).

### 2.4. Quantitative PCR

Quantitative PCR was performed, as we previously published [20]. Briefly, RNA was extracted from the mesothelium and mesothelioma samples using the High Pure FFPET RNA Isolation Kit (Roche). Concentration of the extracted RNA was measured with NanoDrop ND-1000 Spectophotometer (Nano DropTechnologies, Thermo Fisher Scientific, Waltham, MA). Reverse transcription was performed with the High Capacity cDNA Reverse Transcription Kit (Applied Biosystems, Thermo Fisher Scientific) in the ProFlex PCR System Thermal Cycler (Applied Biosystems, Foster City, CA, USA). Gene expression was analyzed using the Cobas z 480 instrument (Roche) according to the manufacturer’s instructions. Relative expression of selected genes was normalized against the endogenous control, *RPLP0*. The following gene expression assays were obtained from the Thermo Fisher Scientific: *POU5F1* (Hs00999632_g1), *NANOG* (Hs04260366_g1), *SOX2* (Hs01053049_s1), *KRT5* (Hs00361185_m1), *WT1* (Hs01103751_m1), *PI3KCA* (Hs00907957_m1), *PIK3CD* (Hs00192399_m1), *AKT1* (Hs00178289_m1), *AKT2* (Hs01086102_m1), *AKT3* (Hs00987350_m1), *BCL2* (Hs00608023_m1) and *RPLP0* (Hs00420895_gH). The fold change in gene expression was calculated with the 2^−ΔΔCt^ method and the presented data were normalized to the mesothelium values.

### 2.5. Mitochondrial Membrane Potential Measurements

Mitochondrial membrane potential (ΔΨm) was analyzed, as we previously published [21]. Briefly, Mero-14 and human aortic endothelial cells (The European Collection of Authenticated Cell Cultures) were grown in 96-well plates. Endothelial cells were used as the control, non-cancerous cell line. A 30 min prior to recording, cells were transferred to the phenol red-free Hank’s Balanced Salt Solution medium and loaded with the 100 nM tetramethylrhodamine ethyl ester (TMRE, Invitrogen, Eugene, OR, USA) for 15 min. After the TMRE washout, the fluorescence intensity was detected at room temperature using the EVOS imaging system and the data were analyzed with the ImageJ software.

### 2.6. Reactive Oxygen Species Measurements

ROS generation was investigated, as we previously published [22]. Briefly, cells were grown in the 96-well plates. After being treated with the antimycin A (2.5 µM, 5 µM and 10 µM) for 3 days, cells were incubated for 30 min in 2 μM CM-H_2_DCFDA (Invitrogen, Eugene, OR, USA). The fluorescence of CM-DCF was acquired by EVOS imaging system at room temperature and analyzed by ImageJ software.

### 2.7. Statistical Analyses

Statistical comparisons were performed using the t-test for two groups or the ANOVA with Tukey post hoc test for multiple group measurements. Differences at *p* < 0.05 were considered significant. Correlation in the gene expression was analyzed using the Pearson correlation coefficient.

## 3. Results

### 3.1. OCT4/POU5F1, NANOG, and SOX2 in Mesothelioma

**Figure 1:**
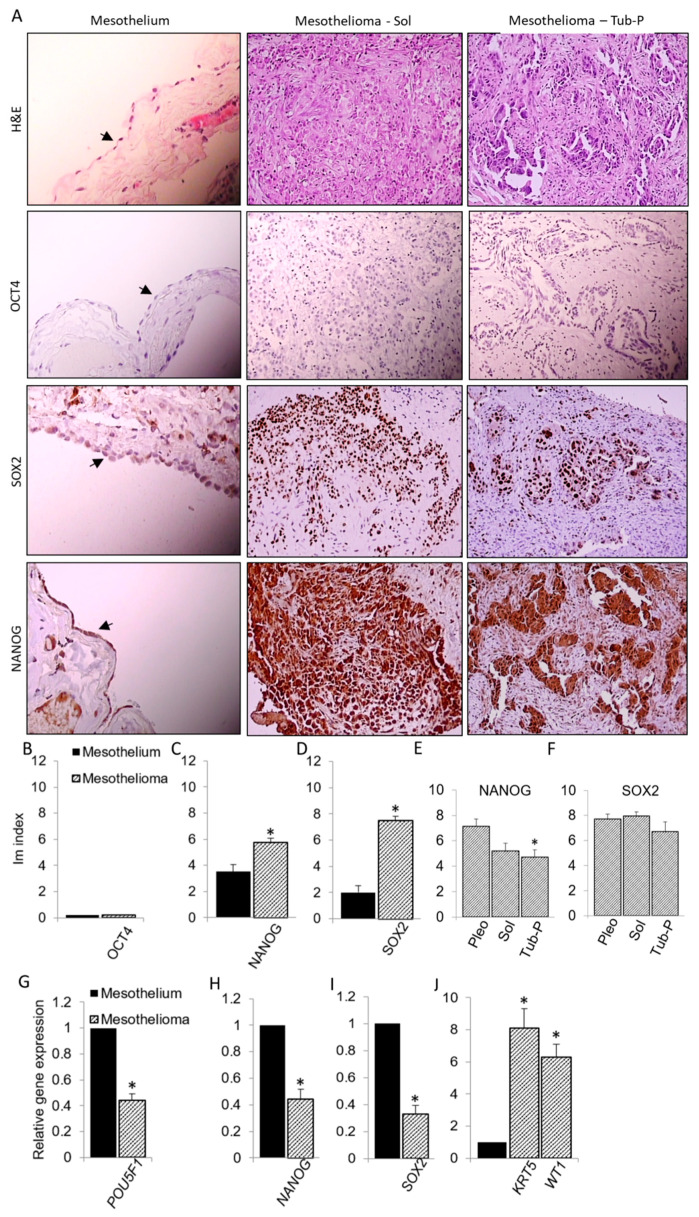
OCT4/*POU5F1*, NANOG and SOX2 expression in mesothelioma. (**A**) Representative images of normal mesothelium and two histological subtypes of mesothelioma, solid (Sol) and tubulopapillary (Tub-P) are shown. Each sample was analyzed by H&E staining and immunohistochemical detection of OCT4, NANOG and SOX2 proteins. Mesothelium images are magnified x400 and arrow marks monolayer of mesothelial cells. Other images are magnified ×200. (**B**–**D**) Summary data of Im index for each protein, representing relative expression of each protein in mesothelium and all mesothelioma samples. (**E**,**F**) Comparison of Im index among three subtypes of mesothelioma, pleomorphic (Pleo), Sol and Tub-P. (**G**–**J**) Shown are summary data of relative expression of pluripotency genes *POU5F1*, *NANOG* and *SOX2*, and mesothelioma marker genes *KRT5* and *WT1*. Data are means ± SEM; * *p* < 0.05 vs Mesothelium or Pleo.

Samples of human normal mesothelium and mesothelioma were analyzed pathohistologically and immunohistochemically to determine the expression of the pluripotency factors, OCT4, NANOG and SOX2 (Figure 1). In mesothelium and mesothelioma, OCT4 protein was not expressed, but NANOG and SOX2 proteins were expressed in portion of cells. SOX2 expression was dominantly nuclear, as previously described [17], while NANOG was expressed in both cytosol and nucleus. Comparison of Im index showed a greater expression of NANOG and SOX2 proteins in mesothelioma than in mesothelium, indicting a dedifferentiation of mesothelioma compared to mesothelial cells from which mesothelioma originates. The expression of SOX2 protein showed no difference among different histological subtypes, while NANOG protein was significantly more expressed in the pleomorphic than in the tubulopapillary mesothelioma. Since the pleomorphic mesothelioma is less differentiated than the tubulopapillary, this indicates that NANOG expression increases with the loss of differentiation of mesothelioma. Unlike OCT4 protein, *POU5F1* gene was expressed in both human mesothelium and mesothelioma, suggesting a posttranscriptional block in its expression in both tissues. Similar to their proteins, *NANOG* and *SOX2* genes were expressed in both mesothelium and mesothelioma. Unlike protein expression, the genes of pluripotency factors, *POU5F1*, *NANOG* and *SOX2*, were less expressed in mesothelioma than in mesothelium. As expected, mesothelioma cancer markers *KRT5* and *WT1* were upregulated in mesothelioma compared to mesothelium.

### 3.2. PI3K, AKT, and BCL2 in Mesothelioma

**Figure 2:**
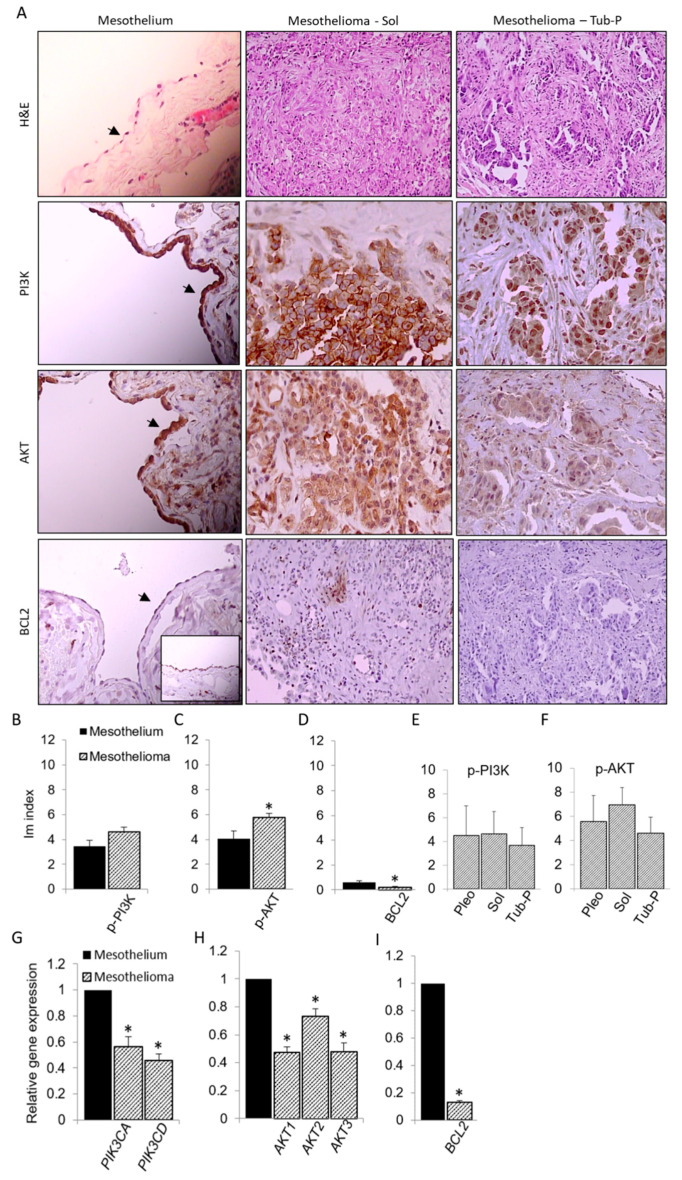
Expression of p-PI3K, p-AKT and BCL2 in mesothelioma. (**A**) Images represent samples of human normal mesothelium and two histological subtypes of mesothelioma, solid (Sol) and tubulopapillary (Tub-P) analyzed by H&E and immunohistochemical staining. All p-PI3K, p-AKT and mesothelium images are magnified ×400 and arrow marks monolayer of mesothelial cells. Other images are magnified ×200. A smaller mesothelioma BCL2 image shows sample with very high expression of BCL2, which is found only in few samples. (**B**–**D**) Summary data of Im index for each protein, representing relative expression of that protein in mesothelium and all mesothelioma samples. (**E**,**F**) Comparison of Im index among three subtypes of mesothelioma, pleomorphic (Pleo), Sol and Tub-P. Relative expression of two PI3K (*PIK3CA* and *PIK3CD*), three AKT (*AKT1*, *AKT2* and *AKT3*) and *BCL2* gene is summarized in (**G**–**I**). Data are means ± SEM; * *p* < 0.05 vs Mesothelium.

PI3K-AKT pathway is important for the malignancy of mesothelioma [23], and it can induce anti-apoptotic BCL2 protein [14]. The mesothelioma samples used for testing OCT4, NANOG and SOX2 expression were also used for investigating the expression of phosphorylated (activated) PI3K (Tyr607; p-PI3K), phosphorylated/activated AKT (Ser473; p-AKT) and BCL2 (Figure 2).

p-PI3K protein was expressed in mesothelium and mesothelioma to the similar extent according to the Im index. p-AKT was expressed in portion of cells in mesothelium and mesothelioma, but overall, it was more extensively expressed in mesothelioma. However, BCL2 protein was expressed only in several mesothelium and mesothelioma samples, with lower expression in mesothelioma. Analysis of the expression of p-PI3K and p-AKT showed no differences among different histological subtypes of mesothelioma. Both PI3K genes, *PIK3CA* and *PIK3CD*, all 3 AKT genes, *AKT1*, *AKT2* and *AKT3*, as well as *BCL2* gene were detected in mesothelium and mesothelioma samples. Unlike PI3K and AKT proteins, all their genes were downregulated in mesothelioma. Only BLC2 exhibited downregulated gene and protein in mesothelioma.

### 3.3. Correlation among Expression of Pluripotency Genes and PI3K, AKT, and BCL2 Genes

**Figure 3:**
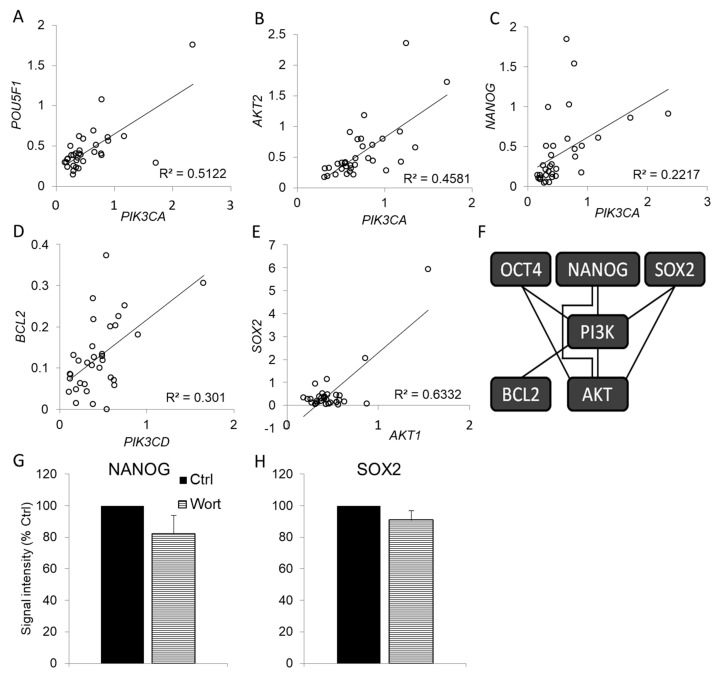
Correlation among expression of genes of pluripotency factors, PI3K-AKT pathway and *BCL2*. (**A**–**E**) Shown are representative correlations among genes expressed in human mesothelioma. (**F**) Genes from Table 1 that exhibit positive correlation of expression are connected in the diagram. (**G** and **H**). Comparison of the expression of NANOG and SOX2 proteins by immunohistochemical quantification in control (Ctrl, *n* = 8) and Mero-14 cells treated with 10 μM wortmannin (Wort, *n* = 8).

Pluripotency factors and PI3K-AKT pathway can regulate each other [15,16,17,18]. The only approach that can test these associations in human mesothelioma samples is the analysis of correlation among the expression of their genes. These results are presented in Table 1 and Figure 3.

Although PI3K-AKT pathway is posttranscriptionally regulated, we also found strong correlations among *PIK3CA* and all 3 AKT genes (*AKT1*, *AKT2* and *AKT3*), and *PIK3CD* and *AKT1*. This suggests that the PI3K-AKT pathway is also regulated at the level of gene expression. Pluripotency factor genes were in correlation with at least one PI3K or AKT gene, but not with anti-apoptotic *BCL2*. This suggests that the expression of all tested pluripotency genes is regulated in association with expression of PI3K and AKT genes. Inhibition of PI3K/AKT pathway with wortmannin in Mero-14 cell did not significantly reduce NANOG or SOX2 protein expression, indicating that pluripotency factors act upstream of PI3K/AKT pathway. *BCL2* was associated with both *PIK3CA* and *PIK3CD* genes.

### 3.4. ΔΨm and ROS Generation in Mitochondria of Mero-14 Cells

Since ROS may modulate the extent of cell differentiation and the expression of pluripotency genes [24,25], and mitochondria-derived ROS depend on the ΔΨm [26], we performed the following experiments to characterize Mero-14 cell mitochondria and their capacity to generate ΔΨm and ROS.

**Figure 4:**
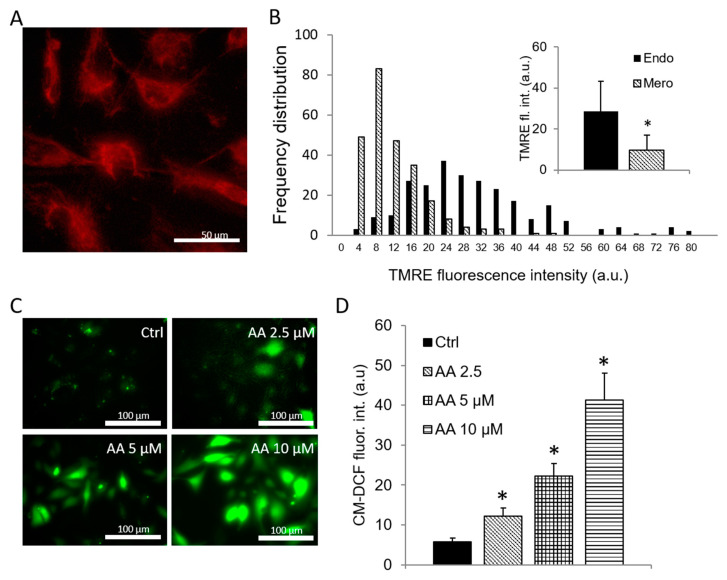
Mitochondrial membrane potential and reactive oxygen species (ROS) generation in human mesothelioma cell line. (**A**) Mitochondria of representative Mero-14 cells are stained with ΔΨm-driven fluorophore TMRE showing presence of functional mitochondria. (**B**) Frequency distribution and average ΔΨm of individual Mero-14 and endothelial (Endo) cells. (**C**) Representative images of Mero-14 cells stained with ROS-sensitive dye CM-DCF in response to increasing antimycin A (AA) concentrations. (**D**) Summary of ROS data (*n* = 7–13/group). Data are means ± SEM; * *p* < 0.05 vs Mero or Ctrl.

Individual Mero-14 cells contained a large number of mitochondria that accumulate TMRE (Figure 4A), indicating that they are functional and capable of generating ΔΨm. However, the average ΔΨm of Mero-14 cell mitochondria was lower than that of non-cancerous human endothelial cell line (Figure 4B). In accordance with this, the analysis of frequency distribution of ΔΨm in 250 individual cells of both cell lines revealed that the Mero-14 cell population was shifted toward lower ΔΨm values than the non-cancerous cell population. However, both populations overlapped considerably, and a good portion of Mero-14 cells contained mitochondria polarized similarly or even more than the median of non-cancerous cell population. Altogether, this indicates that the individual Mero-14 cells contain large number of functional mitochondria, that Mero-14 cells are on average less polarized than the non-cancerous cells, and that a substantial number of Mero-14 cells contain mitochondria with normal ΔΨm.

The baseline CM-DCF signal, an indicator of ROS bioavailability (the difference between formation and degradation of ROS) was low in Mero-14 cells (Figure 4C, D), indicating low baseline ROS bioavailability. However, upon addition of antimycin A, a potent stimulator of ROS production at the respiratory chain complex III, Mero-14 cells responded with a dose-dependent raise in ROS generation that increased eight fold at 10 μM antimycin A. This indicates that in spite of low baseline ROS bioavailability, the mitochondria of Mero-14 cells have a large capacity for ROS generation.

### 3.5. Mitochondria-Derived ROS Induce NANOG Expression

Next we tested whether Mero-14 cells recapitulate the expression pattern of pluripotency factors in human mesothelioma, and examined whether mitochondria-derived ROS affect it, potentially modifying differentiation status of mesothelioma.

**Figure 5:**
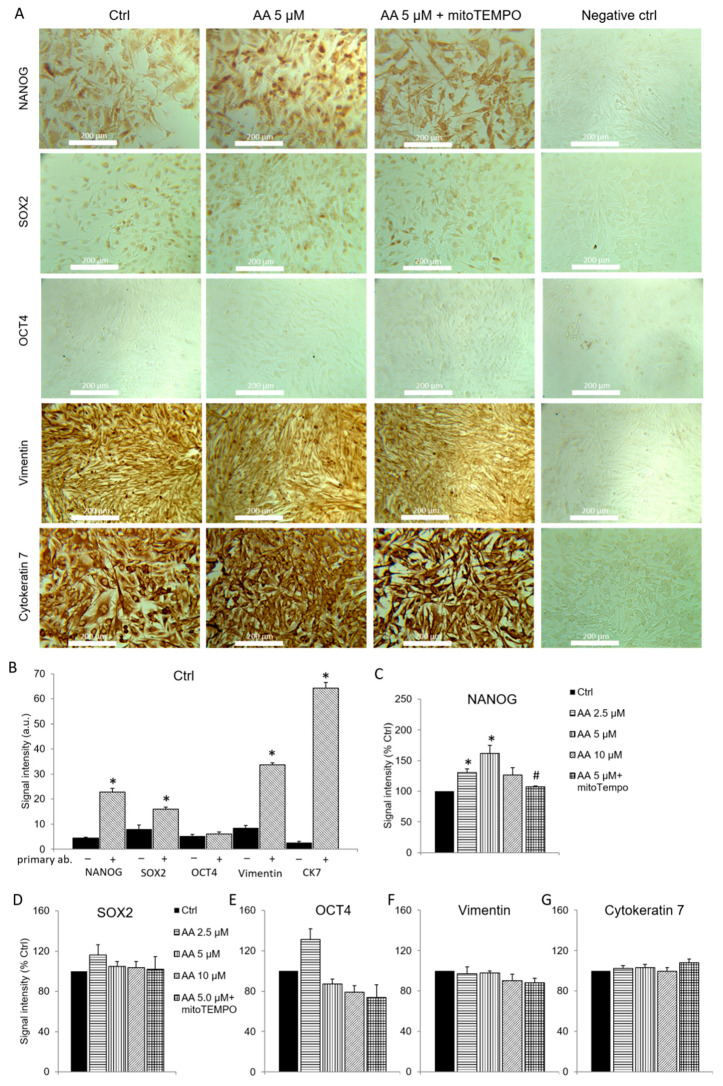
OCT4, NANOG and SOX2 proteins in mesothelioma cell line. (**A**) Representative images of immunocytochemical analysis of protein expression in Mero-14 cells. (**B**) Summary data of protein expression determined by DAB densitometry quantification. The intensity of signal with (+) and without (−) primary antibody is presented and subjected to statistical comparison in control cells (Ctrl). The expression of NANOG (**C**), SOX2 (**D**) and OCT4 (**E**), as well as control mesothelioma markers vimentin (**F**) and cytokeratin 7 (**G**) were analyzed in response to different antimycin A concentrations, with or without mitoTEMPO. Data are means ± SEM; *n* = 8/group. * *p* < 0.05 vs Ctrl.

Immunocytochemical analysis showed that human mesothelioma line exhibited similar pattern of OCT4, NANOG and SOX2 protein expression, and that subcellular localization of NANOG and SOX2 corresponded to human mesothelioma samples (Figure 5). Namely, OCT4 expression was undetectable, while NANOG and SOX2 were expressed almost in all cells. NANOG was expressed in the entire cell and SOX2 predominantly in the nucleus. Application of 2.5 and 5 µM antimycin A, which in previous experiments enhanced mitochondrial ROS formation, dose-dependently increased the expression of NANOG. However, 10 µM antimycin A had no effect on the NANOG expression. Interestingly, cells with extremely high expression of NANOG, which can represent cancer stem cells, were enriched upon ROS-inducing antimycin A treatment (Figure 5A). Mitochondria-targeted ROS scavenger mitoTEMPO abolished the effect of 5 µM antimycin A on NANOG, verifying that mitochondrial ROS induced NANOG expression and thereby partial dedifferentiation of mesothelioma cells. Antimycin A and mitoTEMPO had no effect on the expression of OCT4 and SOX2, nor the expression of control proteins vimentin and cytokeratin 7.

## 4. Discussion

We demonstrated that human mesothelium and mesothelioma express NANOG and SOX2 proteins and *POU5F1*, *NANOG* and *SOX2* genes. Compared to normal mesothelium, mesothelioma exhibited higher expression of NANOG and SOX2 proteins and lower expression of *POU5F1*, *NANOG* and *SOX2* genes. Poorly differentiated histological subtype of mesothelioma had higher expression of NANOG than the subtype with the greater degree of differentiation. While mesothelioma had downregulated all tested PI3K, AKT and BCL2 genes, p-PI3K was unaltered, p-AKT was upregulated and BLC2 protein was downregulated. The expression of PI3K-AKT pathway genes was in association with *POU5F1*, *NANOG* and *SOX2*, and PI3K and BCL2 gene expressions were also in association. Mero-14, human mesothelioma cell line contained functional, but mostly depolarized mitochondria that exhibited large capacity to generate ROS. Individual Mero-14 cells had large number of functional mitochondria, while smaller proportion of Mero-14 cells contained mitochondria with ΔΨm equal to non-cancerous human cells. Mesothelioma cell line recapitulated the expression pattern of OCT4, NANOG and SOX2 proteins in human mesothelioma. Mitochondrial ROS upregulated NANOG, an effect abrogated by the mitochondrial ROS scavenger mitoTEMPO.

This is the first study that extensively tested the expression of OCT4/*POU5F1*, NANOG and SOX2 genes and proteins in human mesothelium and mesothelioma. For the first time, we showed that genes and proteins of pluripotency factors NANOG and SOX2 are expressed in normal mesothelium and malignant mesothelioma. Most of the studies showed the expression of pluripotency genes and proteins in mesothelioma cell lines, but not in human mesothelium or mesothelioma. This includes OCT4 protein expression [27], *POU5F1*, *NANOG* and *SOX2* gene expression in cell lines [28,29] or cancer-initiating cells [30]. Conversely, it has been also shown that *POU5F1*, *NANOG* and *SOX2* are not expressed in mesothelioma cell lines [31]. We found that *POU5F1* was expressed, but OCT4 was not, suggesting its posttranscriptional suppression. Images from Figure 1 show that NANOG and SOX2 staining clearly delineates mesothelioma tissue, which may encourage future studies that would test NANOG and SOX2 as a clinical diagnostic markers in immunohistopathological assays. Although *NANOG* and *SOX2* genes were downregulated in mesothelioma compared to mesothelium, their proteins were upregulated in the cancer. Such opposing results between genes and proteins likely reflect posttranscriptional, translational, and degradation regulation, which ultimately determines protein levels [32]. Since protein expression is directly related to cellular phenotype and therefore more relevant than gene expression, our results indicate that mesothelioma has upregulated NANOG and SOX2 pluripotency factors compared to mesothelium, its tissue of origin, indicating dedifferentiation of mesothelioma. Moreover, poorly differentiated histological subtype, pleomorphic mesothelioma showed greater expression of NANOG than the better-differentiated tubulopapillary subtype. This further demonstrates that increased NANOG expression correlates with poor differentiation of mesothelioma. Others have shown that the high expression pluripotency factors correlates with poor differentiation of different cancer types and poor clinical outcome [33].

The subcellular localization analysis of pluripotency factor protein expression showed that NANOG and SOX2 were expressed in the nucleus and cytosol of human mesothelioma and Mero-14 cells. Topal et al. showed that increased flux of OCT4 and NANOG, but not SOX2, from the nucleus into cytosol is associated with their degradation and differentiation of stem cells [34]. Thus, our observation that NANOG is present in the nucleus of mesothelioma, further corroborates that differentiation processes are inhibited in mesothelioma, rendering mesothelioma a highly undifferentiated cancer.

PI3K-AKT pathway is dysregulated in human mesothelioma [23] and it promotes mesothelioma cell proliferation [35]. Here we showed that PI3K and AKT genes and their phosphorylated forms of proteins are expressed in mesothelium and mesothelioma. Although PI3K and AKT genes were suppressed in mesothelioma, p-AKT protein was upregulated, indicating its increased activity. Thus, our results demonstrate excessive activity of AKT that contributes to the malignant phenotype of mesothelioma. Majority of studies indicate that PI3K-AKT pathway activates OCT4 [15], NANOG [16] and SOX2 [17]. However, OCT4 and NANOG could act upstream of PI3K-AKT pathway [18]. Similarly, a study also showed that upon phosphorylation of OCT4 by AKT, OCT4 dissociated from *AKT1* promotor, stimulating its expression [36]. We showed for the first time in human mesothelioma that PI3K and AKT genes were in positive correlation with the expression of *POU5F1*, *NANOG* and *SOX2*, suggesting positive regulation at the level of gene expression. Thus, our results indicate that increased activity of PI3K-AKT pathway is likely associated with observed upregulation of NANOG and SOX2 in human mesothelioma. Since we showed that wortmannin did not significantly affect the expression of NANOG or SOX2, this suggest that PI3K/AKT pathway does not regulate pluripotency genes in mesothelioma and that pluripotency factors act upstream of it.

*BLC2* expression was in correlation with *PIK3CA* and *PIK3CD*, suggesting association among the expression of these genes. However, BCL2 gene and protein were downregulated in mesothelioma. Although it would be intuitive that, due to its anti-apoptotic activity, low BCL2 expression would suppress cancer, studies show controversial results. Some correlate high BCL2 expression with poor survival [37], while other indicate good prognosis of mesothelioma patients with high BLC2 expression [38]. However, the later study did not determine the BCL2 expression in normal mesothelium, which is important for complete evaluation of those findings. Thus, a downregulation of BCL2 in mesothelioma could promote cancer. However, since BCL2 is poorly expressed in normal mesothelium in our study, it is more likely that it is not important for the mesothelioma.

Immunocytochemical quantification of protein expression in cell culture is highly sensitive and accurate, due to the ability to identify individual cells and normalize the signal to background [39]. The advantage over the time-consuming western blot [40], is that immunocytochemical quantification is not prone to error due to protein crosslinking [41], it allows cell visualization and analysis of cell subpopulations, protein quantification in subcellular compartments and high-throughput experimentation when working with cell culture. This is important when studying SOX2 and NANOG expression, because their subcellular localization can determine the cell fate [34]. However, such quantification of protein expression in tissue slides does not always allow such approach due to the existence of multiple cell layers. Immunohistochemical analysis yields semi-quantitative Im index, which enables simple comparisons [42,43].

Pluripotency factors OCT4 and SOX2 [9] and NANOG [10] are markers of cancer stem cells. Here, we showed that antimycin A treatment enriched cells with very high expression of NANOG (Figure 5A), which can correspond to cancer stem cells. This indicates that mesothelioma reprograming with mitochondrial ROS likely upregulates cancer stem cells. Moreover, by utilizing advantages of immunocytochemical approach, we demonstrated that SOX2 and especially NANOG are expressed in majority of Mero-14 and mesothelioma cells, not just these putative cancer stem cells. This suggests that NANOG and SOX2 are expressed almost in all cell subpopulations of mesothelioma.

In spite of Warburg effect that describes suppressed mitochondrial function in cancer, an increasing number of studies indicate that cancer cell mitochondria function normally and that they have an important role in cancer cell survival [11]. Suppressed mitochondrial metabolism in cancer cells may originate from downregulation of pyruvate dehydrogenase that converts pyruvate to acetyl-CoA that readily enters the citric acid cycle. As a result, mitochondrial would generate lower ΔΨm, less ATP and less ROS [44]. Thus, it was important to determine the capacity of mesothelioma cell mitochondria to generate ΔΨm, as this may dramatically affect their ability to generate ROS. Here we showed that the majority of mesothelioma cells contain functional mitochondria. The frequency distribution of ΔΨm is shifted to lower membrane potentials in the population of mesothelioma cell line than in non-cancerous human cell line, reflecting mitochondrial depolarization in Mero-14 cells, and suggesting Warburg effect in mesothelioma. However, a considerable number of Mero-14 cells contained mitochondria that exhibited equal ΔΨm to non-cancerous human cells, indicating normal mitochondrial metabolism. Such dispersion in the distribution of ΔΨm in human mesothelioma cell line corresponds to variable suppression of mitochondrial metabolisms in subpopulations of cells. Our study is first to characterize the mitochondria of human mesothelioma cells and show that, although depolarized, they are functional and capable of generating ΔΨm.

We demonstrated that, upon stimulation, mesothelioma cell mitochondria generated ROS that upregulated NANOG protein. Our results also indicate that human mesothelioma cells exhibit low baseline ROS bioavailability. This could arise either from low ROS formation or enhanced ROS scavenging capacity, or both. Since Mero-14 cells contain depolarized mitochondria, and depolarized mitochondria generate less ROS [22,26,45], it is plausible that low baseline ROS bioavailability stems from its reduced formation by mitochondria. Proton pumping by respiratory chain complexes is less opposed by lower ΔΨm, which improves coupled electron flux across respiratory chain. As a consequence, electron leak out the respiratory chain and formation of superoxide would be reduced. On the other hand, numerous studies showed that cancer cells can exhibit high levels of ROS scavengers and antioxidants, such as catalase, which can also contribute to observed low baseline ROS formation in mesothelioma cell mitochondria [46]. Importantly, we demonstrated for the first time that mitochondria of human mesothelioma cells have large capacity (at least an eight fold increase upon stimulation) to form ROS. One of our key findings is that mitochondrial ROS induces NANOG protein expression in mesothelioma, which emphasizes the key role of mitochondria in the maintenance of undifferentiated state of human mesothelioma cells. Since upregulation of several pluripotency factors induces cellular reprogramming and dedifferentiation in Yamanaka’s experiment [1], such induction of NANOG in our experiment indicates that partial reprogramming and dedifferentiation of mesothelioma cells can be driven by mitochondrial ROS. Our observation that NANOG is more expressed in human mesothelioma subtype with poor differentiation status further supports the role of NANOG expression in regulation of differentiation status of human mesothelioma. Others have shown that H_2_O_2_ can upregulate *POU5F1*, *NANOG* and *SOX2* in mesothelioma line [28]. However, their experiment relied on non-physiological, exogenous application of H_2_O_2_, while we stimulated endogenous mitochondrial ROS formation, which corresponds better to conditions occurring in the tumor. The upregulation of NANOG by mitochondrial ROS could result from the activation of redox-sensitive transcription factor Nrf2, which binds upstream of *NANOG* gene promoting its expression, and also controls NANOG protein degradation [47]. It has been shown that Nrf2 stimulates proliferation of cancer cells via synthesis of GSH and AKT signaling [48]. Another pathway for NANOG induction by ROS involves HIF-1α. It has been shown in various types of cancer cell lines that ROS can activate HIF-1α, which induced the expression of pluripotency factors [49]. Somewhat surprisingly, we observed that higher (10 μM) antimycin A concentration did not upregulate NANOG. This possibly reflects inhibitory effect on pluripotency factors when ROS act at very high concentrations, which describes a non-linear dose-response curve observed in many physiological systems [50]. Overall, our results indicate an important role of mitochondria and mitochondrial ROS in regulation of differentiation status of human mesothelioma and thereby aggressive behavior of this cancer. In accordance with our results, studies in other cancer and normal cells, showed a crucial role of mitochondrial metabolisms in driving the differentiation process [51,52]. Moreover, cancer cells without mitochondria seem to be unable to form tumors and spread locally and distally [53]. Thus, targeting mitochondrial may represent novel and efficient approach in treating mesothelioma [54].

## 5. Conclusions

Our study demonstrates that NANOG and SOX2 genes and proteins are expressed in human mesothelium and mesothelioma and they may have diagnostic potential. The poor differentiation of human mesothelioma is associated with enhanced expression of NANOG, which can be induced by mitochondrial ROS. Although mostly depolarized, mitochondria of human mesothelioma cells have a large capacity to generate ROS, and thereby trigger partial dedifferentiation and drive reprogramming of mesothelioma. The positive association in the expression of genes of pluripotency factors (*POU5F1*, *NANOG* and *SOX2*) and of PI3K-AKT pathway (*AKT1*, *AKT2*, *AKT3*, *PIK3CA* and *PIK3CD*) indicates positive regulation in their expression, while pluripotency factors act upstream of the PI3K-AKT pathway.

## Figures and Tables

**Table 1:** Correlations among gene expressions.

	POU5F1	NANOG	SOX2	PI3KCA	PI3KCD	AKT1	AKT2	AKT3	BCL2
**POU5F1**	-	0.294 0.092	0.027 0.879	0.711 ** <0.001	0.304 0.080	0.117 0.511	0.454 ** 0.007	0.407 * 0.017	0.263 0.133
**NANOG**	0.294 0.092	-	0.149 0.400	0.456 ** 0.007	0.175 0.323	0.121 0.496	0.565 ** <0.001	0.471 ** 0.005	0.121 0.496
**SOX2**	0.027 0.879	0.149 0.400	-	0.443 ** 0.009	0.228 0.196	0.796 ** <0.001	0.581 ** <0.001	0.496 ** 0.003	0.077 0.666
**PI3KCA**	0.711 ** <0.001	0.456 ** 0.007	0.443 ** 0.009	-	0.511 ** 0.002	0.500 ** 0.003	0.676 ** <0.001	0.508 ** 0.002	0.470 ** 0.005
**PI3KCD**	0.304 0.080	0.175 0.323	0.228 0.196	0.511 ** 0.002	-	0.479 ** 0.004	0.207 0.240	0.275 0.115	0.547 ** 0.001
**AKT1**	0.117 0.511	0.121 0.496	0.796 ** <0.001	0.500 ** 0.003	0.479 ** 0.004	-	0.565 ** <0.001	0.471 ** 0.005	0.274 0.118
**AKT2**	0.454 ** 0.007	0.565 ** <0.001	0.581 ** <0.001	0.676 ** <0.001	0.207 0.240	0.565 ** <0.001	-	0.345 * 0.046	0.268 0.125
**AKT3**	0.407 * 0.017	0.471 ** 0.005	0.496 ** 0.003	0.508 ** 0.002	0.275 0.115	0.471 ** 0.005	0.345 * 0.046	-	0.024 0.891
**BCL2**	0.263 0.133	0.121 0.496	0.077 0.666	0.470 ** 0.005	0.547 ** 0.001	0.274 0.118	0.268 0.125	0.024 0.891	-

Each cell contains Pearson correlation coefficient (up) and a *P* value (down), and the significant correlations are shown in bold (* *p* < 0.05, ** *p* < 0.01).

## Abbreviations

ROS—reactive oxygen species, ΔΨm—mitochondrial membrane potential, TMRE—tetramethylrhodamine ethyl ester.

## References

[1] Takahashi K., Yamanaka S. (2006). Induction of pluripotent stem cells from mouse embryonic and adult fibroblast cultures by defined factors. Cell.

[2] Canfield S.G., Sepac A., Sedlic F., Muravyeva M.Y., Bai X., Bosnjak Z.J. (2012). Marked hyperglycemia attenuates anesthetic preconditioning in human-induced pluripotent stem cell-derived cardiomyocytes. Anesthesiology.

[3] Magee J.A., Piskounova E., Morrison S.J. (2012). Cancer stem cells: Impact, heterogeneity, and uncertainty. Cancer Cell.

[4] Li N., Deng W., Ma J., Wei B., Guo K., Shen W., Zhang Y., Luo S. (2015). Prognostic evaluation of Nanog, Oct4, Sox2, PCNA, Ki67 and E-cadherin expression in gastric cancer. Med. Oncol..

[5] Zhao L., Liu J., Chen S., Fang C., Zhang X., Luo Z. (2018). Prognostic significance of NANOG expression in solid tumors: A meta-analysis. Oncotargets Ther..

[6] Nagata T., Shimada Y., Sekine S., Moriyama M., Hashimoto I., Matsui K., Okumura T., Hori T., Imura J., Tsukada K. (2017). KLF4 and NANOG are prognostic biomarkers for triple-negative breast cancer. Breast Cancer.

[7] Matsuoka J., Yashiro M., Sakurai K., Kubo N., Tanaka H., Muguruma K., Sawada T., Ohira M., Hirakawa K. (2012). Role of the stemness factors sox2, oct3/4, and nanog in gastric carcinoma. J. Surg. Res..

[8] Tsai L.L., Yu C.C., Chang Y.C., Yu C.H., Chou M.Y. (2011). Markedly increased Oct4 and Nanog expression correlates with cisplatin resistance in oral squamous cell carcinoma. J. Oral Pathol. Med. Publ. Int. Assoc. Oral Pathol. Am. Acad. Oral Pathol..

[9] Blum W., Pecze L., Felley-Bosco E., Wu L., de Perrot M., Schwaller B. (2017). Stem Cell Factor-Based Identification and Functional Properties of In Vitro-Selected Subpopulations of Malignant Mesothelioma Cells. Stem Cell Rep..

[10] Jeter C.R., Yang T., Wang J., Chao H.P., Tang D.G. (2015). Concise Review: NANOG in Cancer Stem Cells and Tumor Development: An Update and Outstanding Questions. Stem Cells Dayt. OH.

[11] Fouad Y.A., Aanei C. (2017). Revisiting the hallmarks of cancer. Am. J. Cancer Res..

[12] Nugud A., Sandeep D., El-Serafi A.T. (2018). Two faces of the coin: Minireview for dissecting the role of reactive oxygen species in stem cell potency and lineage commitment. J. Adv. Res..

[13] Luo J., Manning B.D., Cantley L.C. (2003). Targeting the PI3K-Akt pathway in human cancer: Rationale and promise. Cancer Cell.

[14] Pan J.J., Chang Q.S., Wang X., Son Y.O., Liu J., Zhang Z., Bi Y.Y., Shi X. (2011). Activation of Akt/GSK3beta and Akt/Bcl-2 signaling pathways in nickel-transformed BEAS-2B cells. Int. J. Oncol..

[15] Almozyan S., Colak D., Mansour F., Alaiya A., Al-Harazi O., Qattan A., Al-Mohanna F., Al-Alwan M., Ghebeh H. (2017). PD-L1 promotes OCT4 and Nanog expression in breast cancer stem cells by sustaining PI3K/AKT pathway activation. Int. J. Cancer.

[16] Kim J.S., Kim B.S., Kim J., Park C.S., Chung I.Y. (2010). The phosphoinositide-3-kinase/Akt pathway mediates the transient increase in Nanog expression during differentiation of F9 cells. Arch. Pharmacal Res..

[17] Jeong C.H., Cho Y.Y., Kim M.O., Kim S.H., Cho E.J., Lee S.Y., Jeon Y.J., Lee K.Y., Yao K., Keum Y.S. (2010). Phosphorylation of Sox2 cooperates in reprogramming to pluripotent stem cells. Stem Cells Dayt. OH.

[18] Yu K.R., Yang S.R., Jung J.W., Kim H., Ko K., Han D.W., Park S.B., Choi S.W., Kang S.K., Scholer H. (2012). CD49f enhances multipotency and maintains stemness through the direct regulation of OCT4 and SOX2. Stem Cells Dayt. OH.

[19] Masic S., Brcic L., Kruslin B., Sepac A., Pigac B., Stancic-Rokotov D., Jakopovic M., Seiwerth S. (2018). Expression of plakophilin 3 in diffuse malignant pleural mesothelioma. Histol. Histopathol..

[20] Sepac A., Si-Tayeb K., Sedlic F., Barrett S., Canfield S., Duncan S.A., Bosnjak Z.J., Lough J.W. (2012). Comparison of cardiomyogenic potential among human ESC and iPSC lines. Cell Transplant..

[21] Sepac A., Sedlic F., Si-Tayeb K., Lough J., Duncan S.A., Bienengraeber M., Park F., Kim J., Bosnjak Z.J. (2010). Isoflurane preconditioning elicits competent endogenous mechanisms of protection from oxidative stress in cardiomyocytes derived from human embryonic stem cells. Anesthesiology.

[22] Sedlic F., Muravyeva M.Y., Sepac A., Sedlic M., Williams A.M., Yang M., Bai X., Bosnjak Z.J. (2017). Targeted Modification of Mitochondrial ROS Production Converts High Glucose-Induced Cytotoxicity to Cytoprotection: Effects on Anesthetic Preconditioning. J. Cell. Physiol..

[23] Cedrés S., Montero M.A., Martinez P., Martinez A., Rodríguez-Freixinós V., Torrejon D., Gabaldon A., Salcedo M., Ramon Y.C.S., Felip E. (2012). Exploratory analysis of activation of PTEN-PI3K pathway and downstream proteins in malignant pleural mesothelioma (MPM). Lung Cancer Amst. Neth..

[24] Wanet A., Remacle N., Najar M., Sokal E., Arnould T., Najimi M., Renard P. (2014). Mitochondrial remodeling in hepatic differentiation and dedifferentiation. Int. J. Biochem. Cell Biol..

[25] Zhou D., Shao L., Spitz D.R. (2014). Reactive oxygen species in normal and tumor stem cells. Adv. Cancer Res..

[26] Sedlic F., Sepac A., Pravdic D., Camara A.K., Bienengraeber M., Brzezinska A.K., Wakatsuki T., Bosnjak Z.J. (2010). Mitochondrial depolarization underlies delay in permeability transition by preconditioning with isoflurane: Roles of ROS and Ca^2+^. Am. J. Physiol. Cell Physiol..

[27] Kim M.C., Hwang S.H., Kim N.Y., Lee H.S., Ji S., Yang Y., Kim Y. (2018). Hypoxia promotes acquisition of aggressive phenotypes in human malignant mesothelioma. BMC Cancer.

[28] Kim M.C., Cui F.J., Kim Y. (2013). Hydrogen peroxide promotes epithelial to mesenchymal transition and stemness in human malignant mesothelioma cells. Asian Pac. J. Cancer Prev..

[29] Kim H.A., Kim M.C., Kim N.Y., Kim Y. (2015). Inhibition of hedgehog signaling reduces the side population in human malignant mesothelioma cell lines. Cancer Gene Ther..

[30] Ohno Y., Shingyoku S., Miyake S., Tanaka A., Fudesaka S., Shimizu Y., Yoshifuji A., Yamawaki Y., Yoshida S., Tanaka S. (2018). Differential regulation of the sphere formation and maintenance of cancer-initiating cells of malignant mesothelioma via CD44 and ALK4 signaling pathways. Oncogene.

[31] Melotti A., Daga A., Marubbi D., Zunino A., Mutti L., Corte G. (2010). In vitro and in vivo characterization of highly purified human mesothelioma derived cells. BMC Cancer.

[32] Vogel C., Marcotte E.M. (2012). Insights into the regulation of protein abundance from proteomic and transcriptomic analyses. Nat. Rev. Genet..

[33] Ben-Porath I., Thomson M.W., Carey V.J., Ge R., Bell G.W., Regev A., Weinberg R.A. (2008). An embryonic stem cell-like gene expression signature in poorly differentiated aggressive human tumors. Nat. Genet..

[34] Topal T., Kim B.C., Villa-Diaz L.G., Deng C.X., Takayama S., Krebsbach P.H. (2019). Rapid translocation of pluripotency-related transcription factors by external uniaxial forces. Integr. Biol. Quant. Biosci. Nano Macro.

[35] Xu W.F., Liu F., Ma Y.C., Qian Z.R., Shi L., Mu H., Ding F., Fu X.Q., Li X.H. (2019). Baicalin Regulates Proliferation, Apoptosis, Migration, and Invasion in Mesothelioma. Med. Sci. Monit. Int. Med J. Exp. Clin. Res..

[36] Lin Y., Yang Y., Li W., Chen Q., Li J., Pan X., Zhou L., Liu C., Chen C., He J. (2012). Reciprocal regulation of Akt and Oct4 promotes the self-renewal and survival of embryonal carcinoma cells. Mol. Cell.

[37] Honma N., Horii R., Ito Y., Saji S., Younes M., Iwase T., Akiyama F. (2015). Differences in clinical importance of Bcl-2 in breast cancer according to hormone receptors status or adjuvant endocrine therapy. BMC Cancer.

[38] Pillai K., Pourgholami M.H., Chua T.C., Morris D.L. (2013). Does the expression of BCL2 have prognostic significance in malignant peritoneal mesothelioma?. Am. J. Cancer Res..

[39] Poojan S., Kim H.S., Yoon J.W., Sim H.W., Hong K.M. (2018). Determination of Protein Expression Level in Cultured Cells by Immunocytochemistry on Paraffin-embedded Cell Blocks. J. Vis. Exp. JoVE.

[40] Mishra M., Tiwari S., Gomes A.V. (2017). Protein purification and analysis: Next generation Western blotting techniques. Expert Rev. Proteom..

[41] O’Rourke M.B., Padula M.P. (2016). Analysis of formalin-fixed, paraffin-embedded (FFPE) tissue via proteomic techniques and misconceptions of antigen retrieval. Biotechniques.

[42] Kokolakis G., Panagis L., Stathopoulos E., Giannikaki E., Tosca A., Kruger-Krasagakis S. (2008). From the protein to the graph: How to quantify immunohistochemistry staining of the skin using digital imaging. J. Immunol. Methods.

[43] Fedchenko N., Reifenrath J. (2014). Different approaches for interpretation and reporting of immunohistochemistry analysis results in the bone tissue—A review. Diagn. Pathol..

[44] Jin Y., Cai Q., Shenoy A.K., Lim S., Zhang Y., Charles S., Tarrash M., Fu X., Kamarajugadda S., Trevino J.G. (2016). Src drives the Warburg effect and therapy resistance by inactivating pyruvate dehydrogenase through tyrosine-289 phosphorylation. Oncotarget.

[45] Starkov A.A., Fiskum G. (2003). Regulation of brain mitochondrial H2O2 production by membrane potential and NAD(P)H redox state. J. Neurochem..

[46] Glorieux C., Calderon P.B. (2017). Catalase, a remarkable enzyme: Targeting the oldest antioxidant enzyme to find a new cancer treatment approach. Biol. Chem..

[47] Dai X., Yan X., Wintergerst K.A., Cai L., Keller B.B., Tan Y. (2020). Nrf2: Redox and Metabolic Regulator of Stem Cell State and Function. Trends Mol. Med..

[48] Jia Y., Wang H.D., Wang Q., Ding H., Wu H.M., Pan H. (2017). GSH depletion and consequent AKT inhibition contribute to the Nrf2 knockdown-induced decrease in proliferation in glioblastoma U251 cells. Oncol. Rep..

[49] Mathieu J., Zhang Z., Zhou W., Wang A.J., Heddleston J.M., Pinna C.M., Hubaud A., Stadler B., Choi M., Bar M. (2011). HIF induces human embryonic stem cell markers in cancer cells. Cancer Res..

[50] Sedlic F., Kovac Z. (2017). Non-linear actions of physiological agents: Finite disarrangements elicit fitness benefits. Redox Biol..

[51] Mandal S., Lindgren A.G., Srivastava A.S., Clark A.T., Banerjee U. (2011). Mitochondrial function controls proliferation and early differentiation potential of embryonic stem cells. Stem Cells Dayt. OH.

[52] Skoda J., Borankova K., Jansson P.J., Huang M.L., Veselska R., Richardson D.R. (2019). Pharmacological targeting of mitochondria in cancer stem cells: An ancient organelle at the crossroad of novel anti-cancer therapies. Pharmacol. Res..

[53] Tan A.S., Baty J.W., Dong L.F., Bezawork-Geleta A., Endaya B., Goodwin J., Bajzikova M., Kovarova J., Peterka M., Yan B. (2015). Mitochondrial genome acquisition restores respiratory function and tumorigenic potential of cancer cells without mitochondrial DNA. Cell Metab..

[54] Lennon F.E., Cianci G.C., Kanteti R., Riehm J.J., Arif Q., Poroyko V.A., Lupovitch E., Vigneswaran W., Husain A., Chen P. (2016). Unique fractal evaluation and therapeutic implications of mitochondrial morphology in malignant mesothelioma. Sci. Rep..

